# Hepatitis B Vaccine Antibody Response and the Risk of Clinical AIDS or Death

**DOI:** 10.1371/journal.pone.0033488

**Published:** 2012-03-22

**Authors:** Michael L. Landrum, Katherine Huppler Hullsiek, Robert J. O'Connell, Helen M. Chun, Anuradha Ganesan, Jason F. Okulicz, Tahaniyat Lalani, Amy C. Weintrob, Nancy F. Crum-Cianflone, Brian K. Agan, and Infectious Disease Clinical Research Program HIV Working Group

**Affiliations:** 1 Infectious Disease Clinical Research Program, Uniformed Services University, Bethesda, Maryland, United States of America; 2 San Antonio Military Medical Center, Fort Sam Houston, Texas, United States of America; 3 University of Minnesota, Minneapolis, Minnesota, United States of America; 4 Walter Reed Army Institute of Research, Silver Spring, Maryland, United States of America; 5 Naval Health Research Center, San Diego, California, United States of America; 6 Walter Reed National Military Medical Center, Bethesda, Maryland, United States of America; University of California, San Francisco, United States of America

## Abstract

**Background:**

Whether seroresponse to a vaccine such as hepatitis B virus (HBV) vaccine can provide a measure of the functional immune status of HIV-infected persons is unknown.This study evaluated the relationship between HBV vaccine seroresponses and progression to clinical AIDS or death.

**Methods and Findings:**

From a large HIV cohort, we evaluated those who received HBV vaccine only after HIV diagnosis and had anti-HBs determination 1–12 months after the last vaccine dose. Non-response and positive response were defined as anti-HBs <10 and ≥10 IU/L, respectively. Participants were followed from date of last vaccination to clinical AIDS, death, or last visit. Univariate and multivariable risk of progression to clinical AIDS or death were evaluated with Cox regression models. A total of 795 participants vaccinated from 1986–2010 were included, of which 41% were responders. During 3,872 person-years of observation, 122 AIDS or death events occurred (53% after 1995). Twenty-two percent of non-responders experienced clinical AIDS or death compared with 5% of responders (*p*<0.001). Non-response to HBV vaccine was associated with a greater than 2-fold increased risk of clinical AIDS or death (HR 2.47; 95% CI, 1.38–4.43) compared with a positive response, after adjusting for CD4 count, HIV viral load, HAART use, and delayed type hypersensitivity skin test responses (an *in vivo* marker of cell-mediated immunity). This association remained evident among those with CD4 count ≥500 cells/mm^3^ (HR 3.40; 95% CI, 1.39–8.32).

**Conclusions:**

HBV vaccine responses may have utility in assessing functional immune status and risk stratificating HIV-infected individuals, including those with CD4 count ≥500 cells/mm^3^.

## Introduction

Infection with HIV-1 is unsparing, resulting in perturbations in nearly every immune cell type and immune response. How these impairments coalesce to alter immune responses *in vivo* and result in progressive immune depletion and eventually acquired immune definiciency syndrome (AIDS) in untreated HIV-infected persons is unknown. Although CD4 cell count and plasma HIV viral load (VL) are commonly used to assess HIV disease stage [1], [2], [3], they may not provide a complete measure of the functional immunological status of an HIV-infected person, despite receipt of therapy. For example, in the SILCAAT and ESPRIT clinical trials, which evaluated the use of interleukin-2 (IL-2) in subjects receiving highly active antiretroviral therapy (HAART), simply having a higher CD4 cell count as the result of IL-2 treatment did not correspond to a lower risk of experiencing opportunistic disease or death from any cause, suggesting that a greater number of CD4 lymphocytes did not necessarily equate with reconstitution of immune function [4]. Regarding the relationship between CD4 cell counts and VL, Rodriguez, et al. demonstrated that VL predicted less than 10% of observed CD4 cell loss, and others have demonstrated that VL explained less than 5% and 12% of the variability in the rate of CD4 cell loss and rates of progression to AIDS [5], [6]. Therefore, other parameters reflective of immune function, such as responses to vaccines, may serve as important immunological tools to identify subsets of HIV-infected subjects who manifest functional impairment in the immune system, despite high CD4 cell counts or low VL before or after receipt of HAART.

Although not a vaccine in the traditional sense, immune system function in HIV-infected individuals has been probed by eliciting delayed-type hypersensitivity (DTH) skin test reactivity to recall antigens such as candida, mumps, trichophyton, and tetanus toxoid. DTH responses are a strong *in vivo* parameter of cell-mediated immunity (CMI) and predict risk of AIDS, independent of the CD4 count and VL [7], [8], [9], [10], [11], [12]. Investigations in the pre-HAART era within the U.S. military were among the first to establish the validity of cutaneous DTH testing in predicting AIDS progression and staging disease by categorizing DTH responses by the number of positive skin tests [9], [11]. More recently, poor DTH responses have been associated with increased risk of AIDS or death in women receiving HAART [10], and with reduced CD4 cell count reconstitution after HAART initiation [13]. However, despite its potential utility, DTH responses have not been recommended in the clinical setting for several years [14].

One vaccine used commonly in patients with HIV infection is hepatitis B virus (HBV) vaccine [15]. Unlike other vaccines used frequently in HIV-infected patients, such as pneumococcal polysaccharide, tetanus toxoid, and influenza vaccines, assessment of HBV vaccine serologic responses in HIV-infected adults is now recommended by guidelines [1]. Additionally, tests to evaluate serologic responses to HBV vaccine are widely available. Furthermore, as a peptide antigen administered intramuscularly, the development of an antibody response following HBV vaccination requires T-cell help, and is also reflective of other aspects of immune function including antigen presentation, and B-cell function [16], [17], [18], [19]. Therefore, unlike pneumococcal polysaccharide vaccine which is a T-cell independent antigen, HBV vaccine may provide a more complete assessment of B- and T-cell immune function for an HIV-infected individual compared with some of the other vaccines.

While functional responses to both recall and neoantigens have been previously associated with factors known to predict HIV-related disease progression [20], the possible association between HBV vaccine responses and disease progression has been investigated in only one previous study [21]. In that investigation, vaccine responders were less likely to develop persistent generalized lymphadenopathy than non-responders, but firm conclusions were limited as the study included 27 individuals from the pre-HAART era. Therefore, in the current study we sought to evaluate whether HBV vaccine antibody responses predicted the risk of progression to AIDS or death indepdendent of CD4 cell counts, VL, DTH responses, and HAART use in those immunized after HIV diagnosis in a large well-characterized cohort of HIV-infected indivduals.

## Methods

### Study population

The U.S. Military HIV Natural History Study (NHS) is an ongoing, continuous enrollment observational cohort of HIV-infected Department of Defense beneficiaries followed at seven military medical centers in the United States. The comprehensive epidemiology of HBV infection, the effectiveness of HBV vaccination, and factors associated with HBV vaccine responses in this large prospectively followed, continuously enrolling cohort have all been previously described [15], [22], [23], [24], [25]. All adult DoD beneficiaries with a diagnosis of HIV infection followed at a participating site and ability to provide written, informed consent are eligible for participation. Following enrollment patients are seen every six months. Data collected includes demographic information, past and interim medical histories and illnesses, medications, vaccinations, and standard clinical laboratory studies, and for those that died, dates of death. HIV exposure category is not routinely captured, however rates of HIV risk behaviors have been previously reported and intravenous drug use is rare (<3%) [26].

### Ethics Statement

All NHS participants provided written, informed consent, anda pproval for this research was obtained from the Uniformed Services University of the Health Sciences Infectious Disease Institutional Review Board.

### Participant eligibility and definitions

NHS participants were eligible for the current analysis if (1) they were concurrently non-reactive for HBsAg and HBcAb initially following HIV diagnosis, (2) there was a documented date of HBV vaccination after the HIV seropositivity date, and (3) there was a documented hepatitis B virus surface antibody (anti-HBs) measurement 1–12 months after the last dose of vaccine. Participants with a history of HBV vaccination prior to HIV seropositivity were excluded. Individuals were classified as vaccine non-responders (anti-HBs<10 IU/L) or responders (anti-HBs≥10 IU/L) for statistical analyses using the results obtained from commercially available assays utilized at each NHS study site. For individuals without a documented vaccine response, serum repository samples were tested for anti-HBs as previously described [15]. Neither the HBV vaccine dose nor the commercial formulation was captured in the database. The presence of an AIDS-defining illness was defined using 1993 Centers for Disease Control and Prevention criteria [27], with the exception of isolated CD4 cell count <200 cells/mm^3^. DTH skin test responses were assessed and categorized similar to previous investigations by the number of positive skin tests: normal response (the presence of 2 or more positive skin tests), partial anergic response (one positive skin test), and complete anergic response (zero positive skin tests) [9], [11]. HAART was defined as combination antiretroviral therapy with at least three agents from two different drug classes, or the triple nucleoside combination of zidovudine, lamivudine, and abacavir. An HIV seroconverter was defined as an individual with a documented HIV negative screening test followed by reactive screening and confirmatory HIV tests within 5 years.

### Study design and statistical methods

Descriptive statistics were used to examine the characteristics of vaccine responders and non-responders at the time of last HBV vaccination. Medians were reported with inter-quartile ranges (IQR). Proportions were compared with chi-square or Fisher's exact tests; continuous valued variables were compared with general linear models or Kruskal-Wallis tests as appropriate. The number of initial AIDS or death events and the total number of AIDS or death events were summarized for vaccine non-responders and responders. For analyses regarding rates or risk of AIDS or death, follow-up time started on the date of last HBV vaccination. The primary endpoint was the first of an AIDS-defining illness or death within 18 months after the last NHS study visit. Those who did not experience an endpoint were censored at the last NHS study visit. Rates for the primary endpoint were calculated per 100 person-years of follow-up (PY) for vaccine responders and non-responders overall, and for subgroups formed by number of HBV vaccination doses, CD4 cell count, VL, DTH response, and HAART use at the time of last HBV vaccination, and were compared using rate ratios (RR) from Poisson regression. Rates were also calculated for death separately. Univariate and multivariable Cox proportional hazard models were used to evaluate the association of covariates at the time of last HBV vaccination with subsequent development of clinical AIDS or death. All models were stratified by era of HIV diagnosis according to when HAART was introduced into clinical care (i.e., prior to 1996 versus 1996 or later); multivariable models were also adjusted for duration of HIV infection at the time of last HBV vaccination and HIV seroconverter status. Preliminary multivariable models included all baseline covariates associated with clinical AIDS or death with *p*<0.10. Covariates no longer found to be significantly associated with the primary endpoint were then removed in stepwise fashion to derive the final multivariable model. The final model was also repeated for death separately. Two sensitivity analyses were performed for the compound primary endpoint. First, to eliminate confounding due to the use of HAART, analyses were repeated with censoring at the time of HAART initiation. Second, to assess whether HBV vaccine response was associated with AIDS or death among those with high CD4 cell counts, another analysis was done for the subgroup of participants with CD4 cell count ≥500 cells/mm^3^ at the time of last HBV vaccination. Hazard ratios (HR) were reported with 95% confidence intervals (CI). All *p*-values were two-sided with *p*<0.05 considered statistically significant. No adjustments were made for multiple comparisons. All statistical analyses were performed using SAS software (version 9.1, Cary, NC).

## Results

### Participant characteristics

Seven hundred ninety-five participants met study criteria, and of these, 322 (41%) were vaccine responders. (Table 1) The date of administration of the last dose of vaccine was between 1986 to 2010. The median time from HIV seropositivity to receipt of the last HBV vaccination was 2.7 years (IQR, 1.1–6.1). Thirty-nine percent (n = 310) were on HAART at the time of last vaccination. Of participants not on HAART at last vaccination, 74 (46%) responders and 136 (42%) non-responders started HAART after last vaccination, but prior to censoring (*p* = 0.31). Overall, the median CD4 count was 509 cells/mm^3^ (IQR, 372–668) at last vaccination. The median nadir CD4 cell count prior to last vaccination was 352 cells/mm^3^ (IQR, 243–489). Sixty-one percent of vaccinees received ≥3 doses of vaccine. Median VL at the time of last HBV vaccination was 3.0 log_10_ copies/mL (IQR, 1.4–4.1). VL at the time of last vaccination was unknown for 217 (27%) of those included in the study, as this test was not clinically available prior to 1996 when some study participants received HBV vaccination. Cutaneous DTH response at last vaccination was normal for 317 (40%), and unknown for 370 (47%) as this test was not performed at all study sites.

**Table 1:** Participant characteristics overall and by vaccine response at the time of last hepatitis B vaccination.

			Vaccine Response		
Characteristic	N	Overall	Anti-HBs<10 IU/L (N = 473)	Anti-HBs≥10 IU/L (N = 322)	*P*
**Age, median (IQR) years**	795	32.1 (27.2–38.2)	31.6 (27.5–37.9)	32.4 (26.9–38.3)	0.70
**Male gender**	795	695 (87)	422 (89)	273 (85)	0.06
**Ethnicity**	795				0.69
**Caucasian**		360 (45)	220 (47)	140 (44)	
**African American**		315 (40)	184 (39)	131 (41)	
**Other**		120 (15)	69 (15)	51 (16)	
**Year of HIV diagnosis, median (IQR)**	795	1994 (1990–1999)	1992 (1989–1998)	1996 (1992–2001)	<0.001
**Year of last HBV vaccination, median (IQR)**		1999 (1993–2004)	1997 (1993–2002)	2001 (1997–2006)	<0.001
**Years from HIV diagnosis to last HBV vaccination, median (IQR) years**	795	2.7 (1.1–6.1)	2.6 (1.1–5.9)	2.9 (1.1–6.4)	0.29
**Anti-HCV positive**	778	14 (2)	7 (2)	7 (2)	0.58
**Number of vaccinations**	795				0.004
**1–2**		308 (39)	201 (43)	107 (33)	
**3**		269 (34)	161 (34)	108 (34)	
**4–6**		187 (24)	99 (21)	88 (27)	
**≥7**		31 (4)	12 (3)	19 (6)	
**CD4 count, median (IQR) cells/mm^3^**	754	509 (372–668)	455 (326–618)	582 (433–746)	<0.001
**Nadir CD4 count, median (IQR) cells/mm^3^**	697	352 (243–489)	340 (224–475)	376 (261–499)	0.03
**Viral load, median (IQR) log_10_ copies/mL**	577	3.0 (1.4–4.1)	3.5 (2.1–4.3)	2.3 (1.4–3.9)	<0.001
**Prior AIDS-defining illness**	795	45 (6)	28 (6)	17 (5)	0.70
**DTH Response**	795				0.02
**Normal**		317 (40)	203 (43)	114 (35)	
**Partial anergy**		65 (8)	45 (10)	20 (6)	
**Complete anergy**		43 (5)	26 (6)	17 (5)	
**Unknown**		370 (47)	199 (42)	171 (53)	
**Taking HAART**	795	310 (39)	147 (31)	163 (51)	<0.001

NOTE. Data are no. of participants (%) with data available, unless otherwise indicated. IQR, inter-quartile range; Anti-HBs, hepatitis B surface antibody; HCV, hepatitis C virus; DTH, delayed-type hypersensitivity; HAART, highly active antiretroviral therapy.

### Follow-up, outcomes, and rates of events

During a total of 3,872 person-years of observation, 105 (22%) non-responders and 17 (5%) responders experienced a clinical AIDS or death event (*p*<0.001) (Table 2). Of the total 122 initial AIDS or death events, 65 (53%) occurred during 1996 or later. Sixty-seven (14%) non-responders and 11 (3%) responders died during study follow-up (*p*<0.001). The most common AIDS events other than death were *Pneumocystis jiroveci* pneumonia, candidiasis, wasting, *Mycobacterium avium* complex infection and Kaposi sarcoma. Fifty (11%) non-responders had multiple events, compared with 8 (3%) responders (*p*<0.001).

**Table 2:** Number (%) of first AIDS or death events and follow-up time by vaccine response status.

	Anti-HBs<10 IU/L (N = 473)	Anti-HBs≥10 IU/L (N = 322)	*P*
**Sequence of initial event type, N (%)**			<0.001
**No event**	368 (77.8)	305 (94.7)	
**AIDS**	38 (8.0)	6 (1.9)	
**AIDS then death**	41 (8.9)	6 (1.9)	
**Death**	26 (5.5)	5 (1.6)	
**Initial AIDS event by type, N**			
***Pneumocystis jiroveci*** ** pneumonia**	15	2	
**Candidiasis**	14	3	
**Wasting**	9	0	
***Mycobacterium avium*** **-complex infection**	7	0	
**Kaposi sarcoma**	7	0	
**Non-Hodgkin's lymphoma**	4	1	
**Cryptosporidiosis**	4	0	
**AIDS-related dementia**	3	0	
**Cryptococcosis**	3	1	
**Pneumonia**	3	0	
**Cytomegalovirus disease**	2	2	
**Toxoplasmosis**	2	1	
**Progressive multifocal leukoencephalopathy**	2	0	
**Herpes simplex virus**	2	0	
***Mycobacterium tuberculosis***	1	1	
**Histoplasmosis**	1	0	
**Coccidiomycosis**	0	1	
**Total number of events per person, N (%)**			<0.001
**0**	368 (77.8)	305 (94.7)	
**1**	55 (11.6)	9 (2.8)	
**2**	23 (4.9)	6 (1.9)	
**3**	13 (2.8)	1 (0.3)	
**4 or more**	14 (3.0)	1 (0.3)	
**Follow-up time**			
**Total years**	2,236	1,636	
**Median (IQR) years**	3.5 (1.6–6.5)	3.8 (1.7–7.3)	

NOTE. Anti-HBs, hepatitis B surface antibody.

Overall, the unadjusted rate for experiencing an initial AIDS or death event was 4.7 (95% CI, 3.9–5.7) per 100 PY for non-responders compared with 1.0 (95% CI, 0.6–1.7) per 100 PY for responders (*p*<0.001 for the rate ratio comparison). For death events, the rates (95% CI) per 100 PY were 3.4 (2.8–4.1) per 100 PY for non-responders and 0.8 (0.4–1.3) per 100 PY for responders (*p*<0.001). When evaluating rates of the composite end-point of first AIDS or death event within subgroups defined by number of HBV vaccine doses, DTH response, HAART use, CD4 cell count and VL categories at last HBV vaccination, non-responders had significantly higher rates of AIDS or death compared to responders in almost every subgroup (Table 3). The highest rates were seen in non-responders who were also completely anergic (10.1 per 100 PY), those with CD4 cell count <350 at last HBV vaccination (10.6 per 100 PY), those with unknown HIV-RNA status at last vaccination (8.6 per 100 PY), and those who were not on HAART at last HBV vaccination (6.4 per 100 PY). Although the rates of AIDS or death were low for both non-responders and responders who were also on HAART at last vaccination (1.0 and 0.4 per 100 PY, respectively), even among these participants there was a trend for better outcomes in responders (*p* = 0.21).

**Table 3:** Rates for first event of clinical AIDS or death per 100 person-years of follow-up by hepatitis B vaccine response for CD4 cell count, viral load, delayed-typy hypersensitivity response, and highly active antiretroviral therapy subgroups.

Subgroup	Anti-HBs<10 IU/L Rate (95% CI)	Anti-HBs≥10 IU/L Rate (95% CI)	Rate Rato (95% CI)	*P*
**Number of HBV vaccine doses**				
**1–2**	5.6 (4.2–7.5)	2.0 (1.1–3.7)	2.8 (1.4–5.5)	<0.01
**≥3**	4.2 (3.3–5.4)	0.6 (0.3–1.3)	6.8 (3.1–14.9)	<0.001
**CD4 count, cells/mm^3^**				
**<350**	10.6 (8.1–13.8)	2.7 (1.1–6.4)	4.0 (1.6–9.9)	<0.01
**350–500**	3.2 (2.1–4.8)	0.9 (0.3–2.7)	3.7 (1.1–12.5)	0.03
**≥500**	2.6 (1.7–3.9)	0.7 (0.3–1.4)	3.8 (1.6–8.8)	<0.01
**Viral load, copies/mL**				
**≤400**	0.7 (0.3–1.9)	0.0 (0.0- --)	---	1.0
**>400**	3.9 (2.8–5.4)	1.6 (0.8–3.1)	2.4 (1.2–5.0)	0.02
**Unknown**	8.6 (6.7–10.9)	2.0 (1.0–4.1)	4.2 (2.0–8.8)	<0.001
**DTH Response**				
**Normal**	4.1 (3.1–5.4)	1.0 (0.5–2.0)	4.1 (2.0–8.7)	<0.001
**Partial anergy**	8.4 (5.2–13.6)	2.4 (0.8–7.6)	3.5 (1.0–11.8)	0.048
**Complete anergy**	10.1 (5.6–18.2)	0.9 (0.1–6.7)	10.8 (1.4–83.3)	0.02
**Unknown**	3.9 (2.7–5.6)	0.8 (0.3–2.0)	4.6 (1.8–12.0)	<0.01
**Taking HAART**				
**No**	6.4 (5.2–7.8)	1.5 (0.9–2.6)	4.2 (2.4–7.3)	<0.001
**Yes**	1.0 (0.5–2.1)	0.4 (0.1–1.3)	2.4 (0.6–9.2)	0.21
**Overall**	4.7 (3.9–5.7)	1.0 (0.6–1.7)	4.5 (2.7–7.5)	<0.001

NOTE. CI, confidence interval; Anti-HBs, hepatitis B surface antibody; DTH, delayed-type hypersensitivity; HAART, highly active antiretroviral therapy.

### Univariate and multivariate risk of clinical AIDS or death

In univariate analysis, those with non-response to HBV vaccine had a 4-fold increased risk of AIDS or death following the last dose of vaccine compared with those who did respond (HR 4.01; 95% CI 2.37–6.80) (Table 4). Other factors at last vaccination which were associated with increased risk of AIDS or death included lower CD4 cell count at the time of last vaccination, lower nadir CD4 cell count prior to last vaccination, higher or unknown VL at last vaccination, partial or complete anergic DTH response, and not being on HAART at the time of last HBV vaccination.

**Table 4:** Univariate analysis for factors at the time of last hepatitis B vaccination associated with risk of first clinical AIDS or death event, stratified by HIV diagnosis era (pre-1996 versus 1996 or later).

Characteristic	N	Number of Events	Univariate HR (95% CI)	*P*
**Age, per 10 year increase**	795		0.91 (0.72–1.16)	0.45
**Gender**				
**Male**	695	109	1.44 (0.81–2.57)	0.21
**Female**	100	13	Referent	
**Ethnicity**				
**Caucasian**	360	61	Referent	
**African American**	315	44	0.71 (0.48–1.05)	0.09
**Other**	120	17	0.81 (0.47–1.39)	0.44
**Duration of HIV infection, months**				
**<18**	340	40	Referent	
**18–24**	147	29	1.45 (0.90–2.35)	0.13
**>24**	308	53	1.21 (0.79–1.84)	0.38
**Anti-HCV positive**				
**No**	764	115	Referent	
**Yes**	14	1	0.90 (0.13, 6.45)	0.91
**Total no. HBV vaccinations**				
**1–2**	308	55	1.42 (1.00, 2.04)	0.05
**≥3**	487	67	Referent	
**CD4 count, cells/mm^3^**				
**<200**	47	26	6.88 (4.15–11.42)	<0.001
**200–499**	316	59	2.03 (1.34–3.06)	<0.001
**≥500**	391	31	Referent	
**Nadir CD4 count, cells/mm^3^**				
**<350**	343	68	1.83 (1.28–2.63)	<0.01
**≥350**	354	54	Referent	
**Viral load, copies/mL**				
**≤400**	262	4	Referent	
**>400**	315	45	8.15 (2.92–22.69)	<0.001
**Unknown**	218	73	12.29 (4.43–34.14)	<0.001
**Prior AIDS-defining illness**				
**No**	750	112	Referent	
**Yes**	45	10	1.58 (0.83–3.03)	0.17
**DTH Response**				
**Normal**	317	58	Referent	
**Partial anergy**	65	20	2.10 (1.26–3.50)	<0.01
**Complete anergy**	43	12	2.50 (1.34–4.67)	<0.01
**Unknown**	370	32	1.10 (0.71–1.71)	0.68
**Taking HAART**				
**No**	485	112	Referent	
**Yes**	310	10	0.21 (0.11–0.40)	<0.001
**Anti-HBs Response, IU/L**				
**<10**	473	105	4.01 (2.37–6.80)	<0.001
**≥10**	322	17	Referent	

NOTE. N, number of participants with data available; HR, hazard ratio; CI, confidence interval; Anti-HBs, hepatitis B surface antibody; HCV, hepatitis C virus; DTH, delayed-type hypersensitivity; HAART, highly active antiretroviral therapy.

In the final multivariable model for risk of initial AIDS or death event (stratified by HIV diagnosis era and adjusted for HIV seroconversion status and duration of HIV infection prior to last vaccination), having a hepatitis B vaccine non-response was independently associated with a greater than 2-fold increased risk of progression to AIDS or death (HR 2.47; 95% CI, 1.38–4.43) (Table 5; Model 1). Lower CD4 cell count, not being on HAART, higher VL, and partial and complete anergic DTH response all remained associated with an increased risk of AIDS or death. The number of HBV vaccinations and nadir CD4 cell count prior to last vaccination were not included in the final multivariable model because they were not significantly associated with risk of AIDS or death in preliminary multivariable models. Having a non-response to HBV vaccine remained significantly associated with increased risk of AIDS or death in the two sensitivity analyses: with censoring at HAART initiation (Model 2 HR, 3.22; 95% CI, 1.36–7.66); and for the subset of participants with CD4 count ≥500 cells/mm^3^ at time of last HBV vaccination (Model 3 HR, 3.40; 95% CI, 1.39–8.32).

**Table 5:** Multivariate models for factors associated with risk of first clinical AIDS or death event.

	Model 1-Overall^1^		Model 2- Censored at HAART Initiation^2^		Model 3- CD4≥500 at Last HBV Vaccination^3^	
Characteristic	Multivariate HR (95% CI)	*P*	Multivariate HR (95% CI)	*P*	Multivariate HR (95% CI)	*P*
**Ethnicity**						
**Caucasian**	Referent		Referent		Referent	
**African American**	0.61 (0.40–0.91)	0.02	0.62 (0.38–1.01)	0.05	0.67 (0.31–1.48)	0.32
**Other**	0.59 (0.33–1.06)	0.08	0.51 (0.26–0.98)	0.04	0.52 (0.14–1.96)	0.33
**CD4 cell count, per 50 cells/mm** 3 **increase**	0.87 (0.83–0.92)	<0.001	0.83 (0.77–0.88)	<0.001	N/A	
**Viral load, copies/mL**						
**≤400**	Referent		ND^4^		Referent	
**>400**	3.09 (1.03–9.25)	0.04			11.59 (1.22–109.9)	0.03
**Unknown**	4.45 (1.42–13.96)	0.01			10.44 (1.00–109.1)	0.05
**DTH Response**						
**Normal**	Referent		Referent		Referent	
**Partial anergy**	1.77 (1.04–3.01)	0.04	2.44 (1.30–4.56)	<0.01	1.37 (0.39–4.74)	0.62
**Complete anergy**	3.05 (1.56–5.96)	<0.01	4.74 (2.35–9.53)	<0.001	3.31 (0.36–30.45)	0.29
**Unknown**	1.28 (0.78–2.09)	0.34	1.38 (0.79–2.44)	0.26	1.41 (0.50–3.94)	0.52
**Taking HAART**						
**No**	Referent		N/A		Referent	
**Yes**	0.27 (0.12–0.61)	<0.01			1.18 (0.15–9.43)	0.88
**Anti-HBs Response, IU/L**						
**<10**	2.47 (1.38–4.43)	<0.01	3.22 (1.36–7.66)	<0.01	3.40 (1.39–8.32)	<0.01
**≥10**	Referent		Referent		Referent	

1 Model 1 (Overall) includes 754 people with 116 events.

2 Model 2 (Censored at HAART initiation) included 425 participants with 83 events.

3 Model 3 (CD4 ≥500 at last HBV vaccination) included 391 participants with 31 events.

4 Viral load was not included in Model 2 because there were no events in the 23 participants with viral load ≤400 copies/mL.

NOTE. HR, hazard ratio; CI, confidence interval; DTH, delayed type hypersensitivity; HAART, highly active antiretroviral therapy; Anti-HBs, hepatitis B surface antibody; ND, not done; N/A, not applicable. All three models were adjusted for duration of HIV infection at the time of last vaccination (per 6 months), and known HIV seroconversion status. Models 1 and 3 were also stratified for HIV diagnosis era (pre-1996 versus 1996 or later).

## Discussion

We sought to identify a clinically-relevant and easily measured parameter that may give insights into the status of the functionality of the immune system in HIV-infected persons that is independent of the contemporary means used to assess HIV disease stage, namely absolute CD4 T-cell counts. The results of this study indicate that among HIV-infected individuals who receive HBV vaccine after HIV diagnosis, the overall rate of experiencing an initial AIDS or death event was approximately 4-fold higher in vaccine non-responders than responders. Additionally, in a multivariable model, non-response to the vaccine characterized as an anti-HBs of <10 IU/L independently predicted a nearly 2.5-fold increase in the risk of developing clinical AIDS or death. Notably, this association was independent of CD4 cell count, VL, HAART use, duration of HIV infection, HIV seroconversion status, and the previously used marker of immune function, cutaneous DTH to recall antigens. Consistent with these observations, the event rates were significantly different between HBV vaccine non-responders and responders in those with CD4 cell count ≥500 cells/mm^3^ or with normal DTH responses. Collectively, these data indicate that the antibody response to HBV vaccine is tracking a unique aspect of immune function that cannot be optimally captured by assessment of CD4, VL or DTH responses to recall antigens. Furthermore, because HBV response serves as an independent prognosticator of clinical outcome in HIV infected persons, it may have clinical utility in the risk stratification of HIV-infected persons, including those with CD4 cell count ≥500 cells/mm^3^. Finally, as HBV vaccine responses predicted risk of AIDS or death independent of VL these results illustrate further importance of VL-independent determinants of HIV disease outcomes.

The exact mechanisms responsible for non-response to hepatitis B vaccine in HIV-infected individuals are not entirely known. Conceivably, the genetic and immune factors which underlie the non-response to HBV vaccine in HIV-uninfected persons are also present in those with HIV [17], [19], [28], [29], [30]. However, in HIV-infected adults, additional mechanisms may be operative as the rate of non-responsiveness to HBV vaccine is significantly greater. In viremic HIV-infected individuals the interaction between CD40 ligand on CD4 T-cells and CD40 on B-cells (a key interaction for HBV vaccine responsiveness [19]) is impaired [31], [32]. This reduction in helper T-cell function may at least partially explain the poor HBV vaccine responses observed in those with detectable VL. However, this T- and B-cell interaction appears to normalize following HAART [31]. Therefore, the persistently poor HBV vaccine responses seen in those on suppressive HAART [25] suggest that other aspects of immune function required for a response to HBV vaccine remain abnormal in aviremic individuals. Increased CD4 T-cell activation was associated with reduced responses to HBV vaccine and other similar neoantigens in subjects receiving HAART [20], [33]. Additionally, non-response to HBV vaccine in HIV-infected individuals was found to be significantly associated with an increased proportion of regulatory T (T_reg_) cells [34]. T_reg_ cells have also been associated with non-response to HBV immunization and other vaccines in HIV-uninfected adults [35], [36]. Lastly, HBV vaccine non-response may reflect increased underlying immune senescence, which has also been associated with lack of responsiveness to other vaccines in healthy and HIV-infected adults [37], [38], [39], [40].

While the mechanisms discussed above may explain the poor response rates to HBV vaccine in HIV-infected individuals, it is unclear which mechanism(s) may be specifically associated with the risk of AIDS or death. Decreased CMI is the hallmark of HIV-associated immune dysfunction [20], and responses to HBV vaccine are dependent on T-cell help [18], [19], but we found HBV vaccine responses were associated with the risk of AIDS/death independent of CD4 cell count, and a marker of CMI, DTH responses [9], [10]. Furthermore, the independent association between HBV vaccine responses and AIDS/death was observed in the subset of those with CD4 count ≥500 cells/mm^3^. Thus, facets of immune function other than CMI (as assessed by DTH) may also be associated with worse HIV clinical outcomes, and such aspects of immune dysfunction appear to be evident in those with relatively high CD4 cell counts. Immune activation has clearly been associated with HIV progression [41], so perhaps non-response to HBV vaccine serves as a marker of immune activation. Alterations in B-cell function are also well described in HIV and some do persist following HAART [31], but whether such impairments are associated with the risk of AIDS or death is not known. Lastly, while the complete role of T_reg_ cells in HIV pathogenesis and vaccine responsiveness remains undefined [35], immune senescence has been associated with more advanced HIV infection [37] and increased risk of death in non-HIV-infected individuals [42]. Therefore, further investigation of the mechanisms responsible for non-response to HBV vaccine may yield additional insights on aspects of immune function that are associated with clinical outcomes and which remain persistently abnormal or are restored following HAART.

One potential criticism of the current study may be that it included those that received less than the recommended three doses of HBV vaccine. We chose to include those with 1–2 doses of vaccine with the rationale that those who were able to respond to one or two doses of vaccine may have similar, or even better, immune function than those who responded only after 3 or more doses. The profound difference in the rates of AIDS or death for responders and non-responders observed in those with either 1–2 or ≥3 doses (Table 3) suggest inclusion of those with less than 3 doses did not confound the results. Additionally, the number of vaccine doses was not associated with the risk of AIDS or death in preliminary multivariate Cox regression models, so it was not included in the final models. Another criticism may be that we used too wide of a window (1–12 months) to characterize vaccine response, allowing some responders to wane, thus introducing misclassification bias. While this is possible, such bias would only favor the null hypothesis, thus the difference in outcomes we observed is likely valid.

There were additional limitations to the current study. The HIV cohort analyzed in this study is distinct in some aspects when compared with other large HIV cohorts which may limit generalizability of the findings. Such characteristics include enrollment early after HIV infection due to military HIV screening, open access to care in the military health system, and virtually no intravenous drug use [26]. However, these characteristics should only minimize confounding from drug use or differences in access to care, both of which can impact HIV outcomes [43]. The current study does not apply to all HIV-infected individuals. Many patients with HIV infection do not receive HBV vaccine, as approximately 50% may already be HBV infected at the time of HIV diagnosis [22]. Additionally, an increasing proportion of newly diagnosed HIV patients have received HBV vaccine prior to HIV infection [24]. Additionally, the results of the current study may have been confounded as the majority of non-responders were vaccinated in the pre-HAART era, whereas, a higher proportion of responders were vaccinated in the HAART era and receiving HAART at the time of last vaccination. However, the consistency of the results from multivariable models adjusting for era of HIV diagnosis, and HAART use, with the sub-group analysis of patients not receiving HAART suggests that such confounding, if present, was minimal and did not diminish the overall study conclusions. Finally, VL and DTH responses were not available for all participants, and few events occurred in those on HAART and with VL≤400 copies/mL.

In conclusion, the antibody response to HBV vaccine may provide a measure of immune status that cannot be fully captured by assessment of the CD4 count, VL or DTH skin test responses, and as such, may serve as a tool for risk stratification of HIV-infected persons, including those with relatively preserved CD4 cell count. Understanding the reasons for HBV non-response in those with HIV may provide new insights into immune dysfunction during HIV, as well as result in the development of new immune-based therapies.

## References

[1] Centers for Disease Control and Prevention (2009). Guidelines for the prevention and treatment of opportunistic infections in HIV-infected adults and adolescents: recommendations from the CDC, the National Institutes of Health, and the HIV Medicine Association of the Infectious Diseases Society of America.. MMWR.

[2] Mellors JW, Munoz A, Giorgi JV, Margolick JB, Tassoni CJ (1997). Plasma viral load and CD4+ lymphocytes as prognostic markers of HIV-1 infection.. Annals of Internal Medicine.

[3] O'Brien WA, Hartigan PM, Martin D, Esinhart J, Hill A (1996). Changes in plasma HIV-1 RNA and CD4+ lymphocyte counts and the risk of progression to AIDS.. New England Journal of Medicine.

[4] The INSIGHT-ESPRIT Study Group and SILCAAT Scientific Committee (2009). Interleukin-2 therapy in patients with HIV infection.. New England Journal of Medicine.

[5] Rodriguez B, Sethi AK, Cheruvu VK, Mackay W, Bosch RJ (2006). Predictive value of plasma HIV RNA level on rate of CD4 T-cell decline in untreated HIV infection.. JAMA.

[6] Dolan MJ, Kulkarni H, Camargo JF, He W, Smith A (2007). CCL3L1 and CCR5 influence cell-mediated immunity and affect HIV-AIDS pathogenesis via viral entry-independent mechanisms.. Nature Immunology.

[7] Maas JJ, Roos MT, Keet IP, Mensen EA, Krol A (1998). In vivo delayed-type hypersensitivity skin test anergy in human immunodeficiency virus type 1 infection is associated with T cell nonresponsiveness in vitro.. J Infect Dis.

[8] Jones-Lopez EC, Okwera A, Mayanja-Kizza H, Ellner JJ, Mugerwa RD (2006). Delayed-type hypersensitivity skin test reactivity and survival in HIV-infected patients in Uganda: should anergy be a criterion to start antiretroviral therapy in low-income countries?. Am J Trop Med Hyg.

[9] Dolan MJ, Clerici M, Blatt SP, Hendrix CW, Melcher GF (1995). In vitro T cell function, delayed-type hypersensitivity skin testing, and CD4+ T cell subset phenotyping independently predict survival time in patients infected with human immunodeficiency virus.. Journal of Infectious Diseases.

[10] Anastos K, Shi Q, French AL, Levine A, Greenblatt RM (2004). Total lymphocyte count, hemoglobin, and delayed-type hypersensitivity as predictors of death and AIDS illness in HIV-1-infected women receiving highly active antiretroviral therapy.. Journal of Acquired Immune Deficiency Syndromes.

[11] Blatt SP, Hendrix CW, Butzin CA, Freeman TM, Ward WW (1993). Delayed-type hypersensitivity skin testing predicts progression to AIDS in HIV-infected patients.. Annals of Internal Medicine.

[12] Birx DL, Brundage J, Larson K, Engler R, Smith L (1993). The prognostic utility of delayed-type hypersensitivity skin testing in the evaluation of HIV-infected patients.. Journal of Acquired Immune Deficiency Syndromes.

[13] Meroni L, Marchetti G, Gatti N, d'Arminio Monforte A, Galli M (2003). Delayed-type hypersensitivity skin testing can predict CD4 count increase in HIV patients with poor immunologic response to HAART.. J Acquir Immune Defic Syndr.

[14] Aberg JA, Gallant JE, Anderson J, Oleske JM, Libman H (2004). Primary care guidelines for the management of persons infected with human immunodeficiency virus: recommendations of the HIV Medicine Association of the Infectious Diseases Society of America.. Clin Infect Dis.

[15] Landrum ML, Huppler Hullsiek K, Ganesan A, Weintrob AC, Crum-Cianflone NF (2010). Hepatitis B vaccination and risk of hepatitis B infection in HIV-infected individuals.. AIDS.

[16] Bauer T, Jilg W (2006). Hepatitis B surface antigen-specific T and B cell memory in individuals who had lost protective antibodies after hepatitis B vaccination.. Vaccine.

[17] Egea E, Iglesias A, Salazar M, Morimoto C, Kruskall MS (1991). The cellular basis for lack of antibody response to hepatitis B vaccine in humans.. J Exp Med.

[18] Filion LG, Saginur R, Szczerbak N (1988). Humoral and cellular immune responses by normal individuals to hepatitis B surface antigen vaccination.. J Exp Immunol.

[19] Goncalves L, Albarran B, Salmen S, Borges L, Fields H (2004). The nonresponse to hepatitis B vaccination is associated with impaired lymphocyte activation.. Virology.

[20] Valdez H, Smith KY, Landay A, Connick E, Kuritzkes DR (2000). Response to immunization with recall and neoantigens after prolonged administration of an HIV-1 protease inhibitor-containing regimen.. AIDS.

[21] Loke RH, Murray-Lyon IM, Coleman JC, Evans BA, Zuckerman AJ (1990). Diminished response to recombinant hepatitis B vaccine in homosexual men with HIV antibody: an indicator of poor prognosis.. Journal of Medical Virology.

[22] Chun HM, Fieberg AM, Hullsiek KH, Lifson AR, Crum-Cianflone NF (2010). The epidemiology of hepatitis B virus infection in a U.S. cohort of HIV-infected individuals during the last 20 years.. Clinical Infectious Diseases.

[23] Landrum ML, Fieberg AM, Chun HM, Crum-Cianflone NF, Marconi VC (2010). The effect of human immunodeficiency virus on hepatits B virus serological status in co-infected adults.. PLoS One.

[24] Landrum ML, Hullsiek KH, Chun HM, Crum-Cianflone NF, Ganesan A (2011). The timing of hepatitis B virus (HBV) immunization relative to human immunodeficiency virus (HIV) diagnosis and the risk of HBV infection following HIV diagnosis.. American Journal of Epidemiology.

[25] Landrum ML, Huppler Hullsiek K, Ganesan A, Weintrob AC, Crum-Cianflone NF (2009). Hepatitis B vaccine responses in a large U.S. military cohort of HIV-infected individuals: Another benefit of HAART in those with preserved CD4 count.. Vaccine.

[26] Brodine SK, Starkey MJ, Shaffer RA, Ito SI, Tasker SA (2003). Diverse HIV-1 subtypes and clinical, laboratory and behavioral factors in a recently infected US military cohort.. AIDS.

[27] Centers for Disease Control and Prevention (1992). 1993 revised classification system for HIV infection and expanded surveillance case definition for AIDS among adolescents and adults.. MMWR.

[28] Alper CA, Kruskall MS, Marcus-Bagley D, Craven DE, Katz AJ (1989). Genetic prediction of nonresponse to hepatitis B vaccine.. New England Journal of Medicine.

[29] Craven DE, Awdeh ZL, Kunches LM, Yunis EJ, Dienstag JL (1986). Nonresponsiveness to hepatitis B vaccine in health care workers: results of revaccination and genetic typings.. Annals of Internal Medicine.

[30] Wang C, Tang J, Song W, Lobashevsky E, Wilson CM (2004). HLA and cytokine gene polymorphisms are independently associated with responses to hepatitis B vaccination.. Hepatology.

[31] Moir S, Fauci AS (2008). Pathogenic mechanisms of B-lymphocyte dysfunction in HIV disease.. J Allergy Clin Immunol.

[32] Moir S, Ogwaro KM, Malaspina A, Vasquez J, Donoghue ET (2003). Pertubations in B cell responsiveness to CD4+ T cell help in HIV-infected individuals.. PNAS.

[33] Pessoa SD, Miyamoto M, Ono E, Gouvea AFTB, de Moraes-Pinto MI (2010). Persistence of vaccine immunity against hepatitis B virus and response to revaccination in vertically HIV-infected adolescents on HAART.. Vaccine.

[34] del Mar del Pozo Balado M, Leal M, Lagares GM, Mata RC, Lopez-Cortes LF (2010). Increased regulatory T cell counts in HIV-infected nonresponders to hepatitis B virus vaccine.. Journal of Infectious Diseases.

[35] Holmes D, Jiang Q, Zhang L, Su L (2008). Foxp3 and Treg cells in HIV-1 infection and immuno-pathogenesis.. Immunology Research.

[36] Yang Z, Wang L, Niu W, Wu Y, Zhang J (2008). Increased CD4+CD25+FoxP3+ regulatory T cells in the blood of nonresponders after standard hepatitis B surface antigen vaccine immunization.. Clinical Immunology.

[37] Appay V, Almeida JR, Sauce D, Autran B, Papagno L (2007). Accelerated immune senescence and HIV-1 infection.. Experimental Gerontology.

[38] Appay V, Rowland-Jones SL (2002). Premature ageing of the immune system: the cause of AIDS?. Trends in Immunology.

[39] Effros RB (2007). Role of T lymphocyte replicative senescence in vaccine efficacy.. Vaccine.

[40] Lange CG, Lederman MM, Medvik K, Asaad R, Wild M (2003). Nadir CD4+ T-cell count and numbers of CD28+ CD4+ T-cells predict functional responses to immunizations in chronic HIV-1 infection.. AIDS.

[41] Hunt PW (2007). Role of immune activation in HIV pathogenesis.. Current HIV/AIDS Reports.

[42] Effros RB, Dagarag MD, Spaulding CC, Man J (2005). The role of CD8 T cell replicative senescence in human aging.. Immunology Reviews.

[43] Marconi VC, Grandits GA, Weintrob AC, Chun H, Landrum ML (2010). Outcomes of highly active antiretroviral therapy in the context of universal access to healthcare: the U.S. military HIV natural history study.. AIDS Research and Therapy.

